# The AFD-expressed SRTX-1 GPCR does not contribute to AFD thermosensory functions

**DOI:** 10.17912/micropub.biology.001382

**Published:** 2024-11-13

**Authors:** Laurie Chen, Nathan Harris, Piali Sengupta

**Affiliations:** 1 Department of Biology, Brandeis University, Waltham, Massachusetts, United States; 2 Neuroscience Institute, Georgia State University, Atlanta, Georgia, United States

## Abstract

Temperature experience-regulated gene expression changes have been shown to underlie long-term adaptation of the response threshold of the AFD thermosensory neuron pair, and contribute to thermotaxis behavioral plasticity in *C. elegans* . We previously showed that the SRTX-1 GPCR is expressed primarily in AFD and is localized to their sensory endings. Here we find that SRTX-1 levels are regulated by the animal's temperature experience. However, loss or overexpression of *srtx-1* does not affect thermotaxis behaviors or examined temperature-evoked calcium responses in AFD. Our observations suggest that SRTX-1 may modulate AFD responses and behavior under defined temperature conditions, or in response to specific environmental stimuli.

**Figure 1. The SRTX-1 GPCR does not regulate thermosensory responses in AFD:**
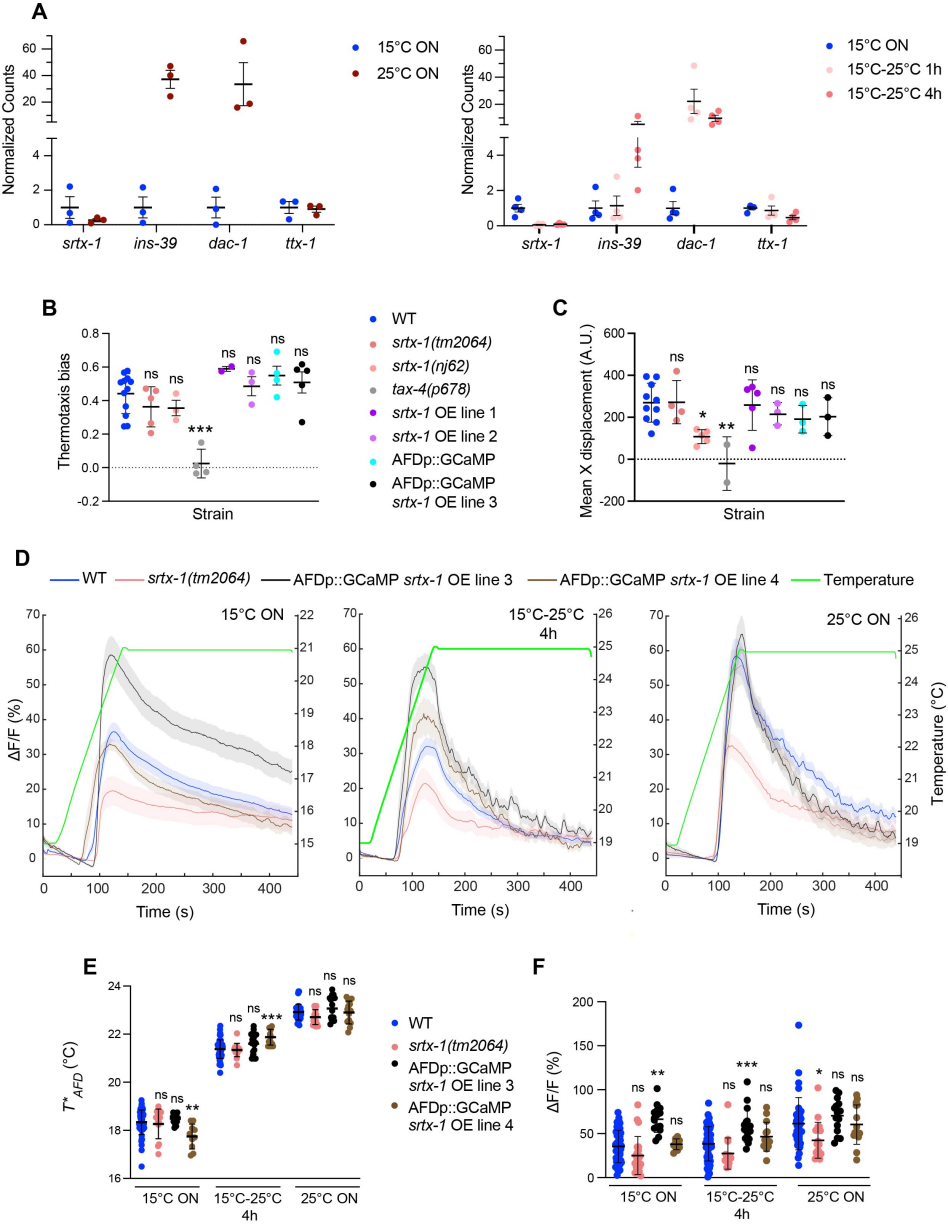
**A)** Normalized read counts of the indicated genes from AFD samples obtained via TRAP-Seq (Harris et al., 2023) after cultivation at the indicated conditions. Animals were raised at 20°C until the L4 stage, then shifted to 15°C or 25°C overnight, or to 25°C for 1-4 hrs (Harris et al., 2023 and unpublished). Normalized counts were calculated in R by DESEQ2 using counts (dds, normalized=TRUE) (Love et al., 2014). *ins-39* encodes an insulin/IGF peptide hormone (Servello et al., 2022); *dac-1* encodes the *C. elegans* ortholog of the DACH1/2 Dachsund transcription factor (Colosimo et al., 2004); *ttx-1* encodes an OTX homeodomain transcription factor (Satterlee et al., 2001). **B)** Mean thermotaxis bias of animals of the indicated genotypes grown at 15°C on a thermal gradient set at 19°C-24°C. Thermotaxis bias: (run duration toward colder side – run duration toward warmer side)/total run duration. Each circle is the thermotaxis bias of a biologically independent assay composed of 15 animals. *** indicates different at P<0.001 from wild-type (one-way ANOVA with Dunnett's correction); ns – not significant. OE – overexpression under the *ttx-1* promoter. AFDp::GCaMP refers to strain DCR3055 (see Table 1). **C)** Quantification of assay endpoints relative to starting position of animals of the indicated genotypes grown at 15°C and shifted to 25°C for 4 hrs prior to being placed on a gradient set at 19°C-23°C. Each circle is the average endpoint distance of all animals in a single assay comprised of 15 animals. * and ** indicate different at P<0.05 and 0.01, respectively from wild-type (one-way ANOVA with Dunnett's correction); ns – not significant. OE – overexpression under the *ttx-1* promoter. AFDp::GCaMP refers to strain DCR3055 (see Table 1). **D)** Average GCaMP6s fluorescence changes in animals of the indicated genotypes grown at the shown temperature conditions in response to a rising temperature ramp (green) at 0.05°C/s. Shaded regions are SEM. **E, F)** T* _AFD_ (E) and maximum response amplitudes (F) calculated from the traces shown in D. Each circle is the measurement from a single AFD neuron. *, **, and *** different from corresponding wild-type at P<0.05, 0.01, and 0.001 respectively (one-way ANOVA with Dunnett's correction); ns – not significant. Vertical and horizontal lines in all scatter plots indicate mean and SD, respectively. Behavioral and calcium imaging data shown are from at least two independent days each.

## Description

The ability to detect and respond appropriately to environmental temperature changes is particularly critical for the survival of poikilotherms which must rely largely on behavioral mechanisms to maintain optimal body temperature (Stevenson, 1985) . The soil-dwelling nematode *C. elegans* exhibits complex experience-dependent navigation behaviors in response to temperature changes. When placed on a thermal gradient within their physiological temperature range, these animals preferentially move towards the temperature they were exposed to prior to the assay (Hedgecock and Russell, 1975) . This behavioral temperature preference can be reset upon exposure to a new temperature for ~3-4 hrs (Hedgecock and Russell, 1975) . Thus, analyses of thermosensory behavioral plasticity in *C. elegans* provides an excellent opportunity to characterize the molecular mechanisms by which experience reshapes neuronal properties to alter output.

Thermotaxis behaviors in *C. elegans* are driven primarily although not exclusively by the AFD thermosensory neuron pair (Mori and Ohshima, 1995; Ryu and Samuel, 2002) . Responses to temperature changes in AFD are detected only above a temperature threshold (referred to as T* _AFD_ ) that is determined by the animal's temperature experience (Clark et al., 2006; Kimura et al., 2004; Ramot et al., 2008) . Shifting animals from a cold to a warm temperature results in a resetting of T* _AFD_ to a higher value within a few minutes via cGMP- and calcium-dependent feedforward and feedback mechanisms (Hawk et al., 2018; Hill and Sengupta, 2024; Ramot et al., 2008; Yu et al., 2014) . However, adaptation of T* _AFD_ to the final value upon a large magnitude temperature upshift requires prolonged hours-long exposure to the warm temperature (Yu et al., 2014) . We recently showed that long-term adaptation of T* _AFD_ requires temperature-regulated changes in the expression of genes, including thermotransduction genes, in AFD (Harris et al., 2023; Yu et al., 2014) , indicating that activity-regulated remodeling of the AFD transcriptome plays a critical role in shaping AFD response plasticity.

Thermotransduction in AFD is mediated via cGMP signaling (Goodman and Sengupta, 2018) , and we previously identified AFD-specific receptor guanylyl cyclases as being both necessary and sufficient for temperature responses in this neuron type (Inada et al., 2006; Takeishi et al., 2016; Wang et al., 2013) . However, AFD also expresses a subset of GPCRs belonging to the larger chemoreceptor family; the functions of these GPCRs are unknown (Colosimo et al., 2004; Taylor et al., 2021; Vidal et al., 2018) . Examination of reporter transgene expression and neuronal transcriptomics data indicate that the *srtx-1* GPCR is expressed primarily in the AFD neurons and localized to their specialized sensory endings, with markedly lower expression in the AWC ^OFF^ olfactory neuron (Colosimo et al., 2004; Nguyen et al., 2014; Taylor et al., 2021) . We previously showed that basal activity of the AWC neurons is increased in *srtx-1* mutants (Biron et al., 2008) , but the role of SRTX-1 in regulating AFD temperature responses remains to be fully described.

Analysis of the temperature-regulated gene expression program in AFD via translating ribosome affinity purification (TRAP) (Harris et al., 2023) showed that expression levels of *srtx-1* were decreased in animals grown overnight at 25ºC as compared to levels upon growth at 15ºC ( Figure 1A ). Moreover, *srtx-1* expression decreased rapidly upon a temperature upshift from 15ºC to 25ºC for 1-4 hrs ( Figure 1A ; N.H. and P.S., in preparation). We previously identified additional genes which also exhibit similar expression changes upon a temperature upshift (Harris et al., 2023) . This pattern of expression change is in contrast to the rapid or delayed upregulation of a subset of genes such as *dac-1* and *ins-39* ( Figure 1A ) whose altered expression has been shown to underlie different aspects of AFD-driven physiological and behavioral plasticity upon a temperature upshift (Harris et al., 2023; Servello et al., 2022) .

We determined whether AFD-driven thermotaxis behaviors are altered in *srtx-1* mutants. Wild-type animals grown at 15ºC move towards colder temperatures (negative thermotaxis) on a spatial thermal gradient (Hedgecock and Russell, 1975) . Both *srtx-1 ( tm2064 )* and *srtx-1 ( nj62 )* mutants exhibited robust negative thermotaxis behavior under these conditions ( Figure 1B ). Wild-type and *srtx-1 ( tm2064 )* mutants also moved similarly towards warmer temperatures (positive thermotaxis) upon being shifted from 15ºC to 25ºC for 4 hrs, although *srtx-1 ( nj62 )* animals exhibited defects in this behavior ( Figure 1C ). Since both *srtx-1 ( tm2064 )* and *srtx-1 ( nj62 )* are predicted to be null alleles (Biron et al., 2008) , the observed behavioral defects in *srtx-1 ( nj62 )* animals may be due to background mutations. Animals mutant for the *tax-4* cyclic nucleotide-gated channel gene essential for temperature responses in AFD failed to perform either negative or positive thermotaxis ( Figure 1B -C). Since *srtx-1* expression is decreased upon a temperature upshift, we also examined whether overexpression of *srtx-1* under a temperature-insensitive promoter would affect thermotaxis. However, neither negative nor positive thermotaxis was affected in animals overexpressing *srtx-1* under an AFD-specific *ttx-1* promoter ( Figure 1B -C) (Satterlee et al., 2001) . We conclude that SRTX-1 does not contribute to AFD-driven thermotaxis behaviors under the examined conditions.

To further assess whether SRTX-1 regulates AFD thermosensory responses, we examined temperature-evoked calcium responses in AFD. T* _AFD_ and response amplitudes were quantified in animals expressing the genetically encoded calcium sensor GCaMP6s in AFD and subjected to a rising temperature ramp from either 15ºC-21ºC for animals grown at 15°C overnight, or from 19-25°C for animals grown either at 15°C overnight and shifted to 25°C for 4 hrs prior to imaging, or at 25°C overnight. T* _AFD_ was unaltered in *srtx-1 ( tm2064 )* mutants under any examined temperature conditions, whereas overexpression of *srtx-1* in AFD resulted in a minor decrease in T* _AFD_ in only one of two overexpressing lines in a subset of conditions ( Figure 1D -E). Response amplitudes were slightly decreased in *srtx-1* mutants only upon overnight growth at 25ºC ( Figure 1D, 1F), whereas amplitudes were significantly increased in one of two *srtx-1* overexpressing lines (line 3) upon overnight growth at 15ºC or upon a 15ºC to 25ºC shift for 4 hrs ( Figure 1D, 1F). However, the increased response amplitude in this overexpressing line did not alter either negative or positive thermotaxis behavior ( Figure 1B -C). We infer that neither loss nor overexpression of *srtx-1* alters AFD temperature responses sufficiently to affect thermotaxis behavior.

Results described here suggest that despite being localized to the AFD sensory endings, and being regulated by a specific temperature experience, the SRTX-1 GPCR does not appear to significantly modulate AFD temperature responses or AFD-driven thermotaxis behaviors under the conditions examined in this study. It is possible that a role for this GPCR may be uncovered under temperature stimulus conditions that we have not examined here. Alternatively, SRTX-1 may act redundantly with other AFD-expressed GPCRs to regulate neuronal functions. The sensory endings of AFD are embedded in the processes of the amphid sheath (AMsh) glial cell (Doroquez et al., 2014; Ward et al., 1975) . The AMsh glia form a microdomain around the AFD sensory endings, and glial-neuron communication plays an important role in shaping AFD sensory ending structure and function (Bacaj et al., 2008; Ray et al., 2024; Singhvi et al., 2016) . Under specific environmental conditions, a glia-produced molecule may interact with SRTX-1 , and/or SRTX-1 may respond to one or more environmental chemicals to modulate temperature responses in AFD. Identification of the SRTX-1 ligand(s) will allow us to further characterize the role of this GPCR in regulating AFD response properties.

## Methods

***C. elegans* strains**

Worms were grown on *E. coli* OP50 bacteria, and 1 day-old adults were examined in all experiments. Strain genotypes were confirmed by PCR-based analyses and/or visual examination for the presence of the fluorescent reporter. The *ttx-1* p *:: srtx-1* plasmid was injected at 10 ng/µl together with the *unc-122* p:: *dsRed* co-injection marker at 50 ng/µl to generate overexpressing transgenic lines. At least two independent transgenic lines were examined for each injected construct.

**Molecular biology**

The *ttx-1* p *:: srtx-1* plasmid was constructed by amplifying *srtx-1* cDNA from a plasmid (gift from Aakanksha Singhvi) and using standard restriction enzyme-based cloning to insert the cDNA into a plasmid containing *ttx-1* promoter sequences driving AFD-specific expression. The plasmid was verified by sequencing.

**Thermotaxis behavior**

Thermotaxis behaviors were performed as described (Yeon et al., 2020; Harris et al., 2023; Hill and Sengupta, 2024). L4 larvae were grown at 15°C overnight prior to performing negative thermotaxis assays. Adult animals grown at 15°C were shifted to 25°C for 4 hrs prior to performing positive thermotaxis assays. Negative thermotaxis assays were performed by placing 15-20 animals on a 10 cm circular NGM agar plate with the gradient set at 19°C-24°C. Animals were recorded at 1 Hz for 35 min using PixeLink CCD cameras and videos were analyzed using custom scripts as described (Beverly et al., 2011; Yu et al., 2014). Positive thermotaxis assays were performed by placing 20-30 animals on a 22.5 cm square NGM agar plate with the gradient set at 19-23°C. Animal movement was imaged at 2 fps for 60 min, and analyzed using custom scripts as described (Luo et al., 2014) . Behavioral assays were quantified over at least two independent days for each genotype and condition.

**Calcium imaging**

Measurements of temperature-evoked intracellular calcium dynamics in AFD were performed as described previously (Harris et al., 2023; Hill and Sengupta, 2024) . Videos of calcium changes in the AFD soma were acquired using a Zeiss 10X air objective on a Zeiss Axioskop2 Plus microscope with a digital camera (Hamamatsu Orca). Calcium imaging data were analyzed using custom scripts, and T* _AFD_ and response amplitudes were quantified as described (Harris et al., 2023; Hill and Sengupta, 2024) . Calcium responses were quantified over at least two independent days per genotype and condition.

**Statistical analyses**

Statistical analyses were performed using PRISM software. The number of animals examined in each experiment and the statistical tests used are described in the Figure Legend.

## Reagents

**Table 1.** Strains used in this work.

**Table:** 

**Strain**	**Genotype**
Wildtype	N2 (Bristol)
PR678	*tax-4 ( p678 ) *
PY5614	*srtx-1 ( tm2064 ) *
IK646	*srtx-1 ( nj62 ) *
DCR3055	*wyIs629 [ gcy-8* p *::GCaMP6s; gcy-8* p *::mCherry; unc-122* p *::gfp]*
PY12700	*srtx-1 ( tm2064 ); wyIs629 [ gcy-8* p *::GCaMP6s; gcy-8* p *::mCherry; unc-122* p *::gfp]*
PY12701	*oyEx757 [ ttx-1* p *:: srtx-1 ; unc-122 p::dsRed]* line 1
PY12702	*oyEx758 [ ttx-1* p *:: srtx-1 ; unc-122 p::dsRed]* line 2
PY12703	*wyIs629 [ gcy-8* p *::GCaMP6s; gcy-8* p *::mCherry; unc-122* p *::gfp] oyEx759 [ ttx-1* p *:: srtx-1 ; unc-122 p::dsRed ]* line 3
PY12704	*wyIs629 [ gcy-8* p *::GCaMP6s; gcy-8* p *::mCherry; unc-122* p *::gfp] oyEx760 [ ttx-1* p *:: srtx-1 ; unc-122 p::dsRed]* line 4
